# Nurses’ innovative use of information systems: a narrative review

**DOI:** 10.3389/fpubh.2026.1808207

**Published:** 2026-05-25

**Authors:** Yunyu Guo, Yueying Jiang, Yuan Zhao, Yue Zhao, Dandan Chen, Yuping Zhang, Jing Shao, Hui Zhang, Leiwen Tang

**Affiliations:** 1 Department of Nursing, The Second Affiliated Hospital Zhejiang University School of Medicine, Hangzhou, Zhejiang, China; 2 School of Nursing and Institute of Nursing Research, Zhejiang University School of Medicine, Hangzhou, Zhejiang, China; 3 Department of Nursing, Guizhou Provincial People’s Hospital, Guiyang, Guizhou, China

**Keywords:** innovative use, narrative review, nurse, Nursing Information Systems, use behavior

## Abstract

**Background:**

Despite the widespread adoption of Nursing Information Systems (NIS), nurses often remain positioned as passive users, which limits the potential for system-driven care transformation.

**Objectives:**

This narrative review synthesizes evidence on nurses’ innovative use behaviors in NIS to identify actionable pathways for empowering nurses as co-designers of digital health solutions.

**Methods:**

A synthesis of peer-reviewed literature was conducted using PubMed, Web of Science, CNKI, Wanfang, and VIP. Studies were selected based on relevance to nurses’ roles in NIS innovation following the SANRA principles.

**Results:**

Nurses are key drivers in NIS innovation, shifting from passive users to proactive contributors to digital health. Evidence shows nurse-led initiatives improve patient safety, documentation, and care efficiency. Theories explain how nurses adapt NIS to clinical needs, especially in resource-limited settings. However, barriers remain, including inconsistent policies, inadequate digital training, and unequal system access.

**Discussion:**

Advancing NIS innovation may require a policy shift from basic functional training toward structural empowerment. Policymakers and healthcare leaders should consider positioning nurses as system co-designers, providing protected resources and formal incentive mechanisms to foster a sustainable digital nursing ecosystem.

## 1 Background

Driven by the policy of *Digital health transformation and nursing practice* (1), deepening the construction of nursing informatization has become the inevitable path for the high-quality development of nursing. As the cornerstone of nursing informatization, the Nursing Information System (NIS) encompasses a diverse range of functional types, extending from clinical nurse workstations to specialized management platforms for patient information, quality control, human resources, and online education. It features rapid collection, large-scale storage, intelligent processing and transmission of various clinical nursing and nursing management business data and information (2). Nurses, as the core users of the NIS, their innovative use behavior refers to the high-level behavior that goes beyond the execution of the basic functions of the NIS, explores the potential functions and values of the NIS, and actively participates in the reform and research and development of the system (2, 3), which not only optimizes the nursing process, enhances the quality and efficiency of nursing, but also promotes the iterative upgrade of system effectiveness to meet the continuous emergence of new nursing demands in the future.

However, the current role of international nurses in NIS utilization remains primarily at the basic implementation level, focusing on repetitive, routine clinical tasks, while innovative use behaviors have received insufficient attention and show a notable lag (2, 4). This is largely because the concept of innovative use behaviors originated in enterprise management (3). Consequently, its conceptual evolution and adaptation within the highly specialized and high-pressure clinical nursing environment remain subject to cognitive bias (5–7). Furthermore, the lack of a consensus definition in nursing contexts has resulted in fragmented practical summaries (2, 8). Due to the limitations of existing assessment tools and evaluation frameworks, the underlying mechanisms of its factors remain obscured, making it difficult to provide evidence-based recommendations for subsequent policy development and clinical leadership (2, 9–11). Therefore, this article conducts a review on the concept, current practices, assessment tools, influencing factors and improvement strategies of innovative usage behavior of NIS, aiming to provide references for enhancing the level of innovative usage behavior of NIS, stimulating nurses’ active innovation potential, and promoting the construction of smart nursing.

## 2 Methods

A narrative synthesis was conducted to explore nurses’ innovative use of NIS following the Scale for the Assessment of Narrative Review Articles (SANRA) (12) principles, which primarily guided the review’s reporting structure and synthesis logic (Appendix 1). Because a global consensus definition of this concept is lacking, we intentionally employed a broad, systematic search strategy to capture diverse and fragmented clinical practices. We searched PubMed, Web of Science, CNKI, Wanfang, and VIP from database inception to 2025. The search strategy employed specific Boolean operators combining three core categories: (1) “Nurse,” (2) “Nursing Information Systems,” and (3) “innovative usage behavior” (Appendix 1, Table A1). To ensure high relevance and minimize noise, keywords were restricted to the [Title/Abstract] field, with strategy adjustments tailored to each database’s syntax requirements. To ensure evidence quality and manage the feasibility of the extensive dataset, studies were screened against predefined inclusion and exclusion criteria (Appendix 1, Table A2) through a standardized workflow. Following automated deduplication, two researchers independently conducted a rapid title/abstract screening to efficiently filter out irrelevant records, followed by a rigorous full-text evaluation. Any discrepancies were resolved by a third senior researcher (L. T.) to ensure screening accuracy. Data were then extracted using a standardized template capturing study design, functional NIS types, and specific innovative behaviors. We employed a hybrid thematic synthesis approach to integrate the evidence. Regarding the heterogeneity of included studies (e.g., empirical research, practice reports, and theses), evidence was synthesized using a contribution-led approach rather than hierarchical weighting. Specifically, empirical studies and reviews provided the theoretical foundation for determinants and assessment tools, while practice reports and theses were utilized to enrich the descriptive mapping of specific innovative behaviors. Operationally, four primary thematic domains—conceptualization, current practice, assessment tools, and determinants—were deductively established based on the review’s overarching research objectives. Within these domains, particularly concerning current practices, the extracted data were processed through a multi-stage operational workflow: (1) descriptive coding of specific nurse-system interactions; (2) inductive clustering of these codes into sub-themes; and (3) deductive mapping of these sub-themes onto the “Exploitation-Exploration” dual-framework. Finally, all derived themes were iteratively refined and validated through expert panel discussions involving nursing managers, clinical nurses, and informatics specialists to ensure multi-dimensional interpretive validity and clinical relevance.

## 3 Results

### 3.1 Characteristics of included studies

From an initial pool of 20,126 identified records, 15,841 unique titles were screened and 246 full-text articles were rigorously evaluated, resulting in 37 studies that met the strict inclusion criteria for final synthesis. These studies were published between 1993 and 2025, with a significant increase in research output observed after 2020. The geographic distribution is predominantly centered in China (including Taiwan, *n* = 24), followed by the United States (*n* = 5) and the United Kingdom (*n* = 2), with additional contributions from Canada, Finland, Germany, South Korea, Saudi Arabia, Egypt, Thailand, Italy, Spain, and Iran. The evidence base encompasses a diverse array of research designs: system design, clinical application reports, and practice-oriented analyses (*n* = 15); empirical research including qualitative, cross-sectional, and latent profile analyses (*n* = 9); scale development and psychometric validation (*n* = 3); and a variety of comprehensive reviews including systematic, umbrella, and realist syntheses (*n* = 6). Additionally, the synthesis incorporates master’s theses, theoretical models, and expert viewpoint studies (*n* = 4).

### 3.2 Conceptualization

The innovative usage behavior of information systems originated in the field of enterprise information management (3). Its core lies in those users, in order to meet their work requirements, actively explore and fully utilize the potential functions of the system, and even creatively practice and break through the preset functional framework of the system. Over the past few decades, the construction of nursing informatization has been continuously advanced, but nurses’ use of NIS has been less than satisfactory (2). Due to the current usage situation dominated by the execution of basic operations in a conventional way, the output and transformation of the system have been hindered. To solve this difficult problem, Chinese scholar Xin Wen first focused the research perspective on the innovative use behavior of information systems on the nurse group in 2017 and proposed a two-dimensional framework of expansion and exploration (9). With the deepening of related research, scholars have integrated its concepts based on this two-dimensional framework (2, 3), that is, on the basis of proficiently mastering and applying the basic functions of the NIS, nurses actively learn and expand other functions and usages of the system, and explore innovative nursing solutions with the help of diversified technologies to achieve the optimization of nursing services. The exploitation dimension emphasizes nurses’ in-depth utilization of existing NIS functions and their discovery and combined application of latent capabilities, whereas the exploration dimension foregrounds nurses’ integration of novel tools and technologies, development of new software or systems, and implementation of breakthrough practices and methods during system use. This conceptual framework provides a theoretical anchor point for the connotation evolution and future research of the innovative usage behavior of nurses’ NIS, but it still needs to be further clarified, and a consensus definition established.

### 3.3 Current practices

To provide a structured overview of the synthesized findings, Table 1 summarizes the specific innovative use behaviors across various nursing domains and their corresponding evidence sources.

**Table 1:** Synthesis of nurses’ innovative use behaviors in current practices.

Domain	Sub-domain	Dimension	Specific innovative use behaviors	Key Outcomes/evidence	Sources
Specialized nursing care	/	Exploitation	Embedding evidence-based clinical pathways for enterostomy into the existing NIS.	Doubled adherence to critical nursing interventions.	Pei et al. (13)
		Exploration	Developing bespoke “smart wards” with AI-driven risk alerts and consultation platforms.	Enhanced patient safety and specialty workflow efficiency.	Liu et al. (14) and Guo et al. (15)
Nursing management	Human-resource management	Exploitation	Mining NIS barcode data and operational logs for staffing and workload assessment.	Achieved personalized, data-driven scheduling and optimized staffing.	Nantsupawat et al. (16) and Knox et al. (18)
	Standardized training and assessment	Exploitation	Integrating skill management modules and flexible assessment mechanisms into NIS.	Realized flexible, autonomous, and diverse assessment modes; enabled managers to utilize real-time feedback data for decision-making.	Zhou et al. (20)
		Exploration	Achieving a breakthrough by integrating virtual reality (VR) technology into the NIS framework.	Improved teaching engagement through gamified clinical scenarios.	Zhao et al. (19)
	Workflow and quality management	Exploitation	Designing office systems using standard databases (access); implementing electronic triage support and risk calculators for unplanned extubation.	Improved triage accuracy; reduced administrative workload through standardized office automation; provided data-driven clinical decision support.	Agnihotri et al. (24), Zhu et al. (25), and Wang et al. (26)
		Exploration	Developing intelligent transfusion and multi-terminal quality control systems.	Automated planning and evaluation of departmental activities.	Dai et al. (22) and Wan et al. (23)
Internet-enabled services	Home-based care	Exploration	Developing and constructing dedicated “Internet + Nursing” platforms to integrate online-offline service workflows.	Achieved a breakthrough in service boundaries; Realized full-process monitoring and safety management for home visits.	Dukhanin et al. (28) and Cui et al. (27)
	Nurse-led outpatient clinics	Exploitation	Utilizing NIS functions to support case management, patient education, and follow-up in specialized nursing clinics.	Enhanced the efficiency of specialist nurses in managing chronic diseases and pediatric epilepsy.	Yu et al. (29), Liu et al. (30), and Lyu and Shen (31)
	Virtual wards	Exploration	Establishing decentralized care units with 24 h remote monitoring to replace physical beds.	Created a bed-free clinical environment; optimized hospital resource allocation.	Westby et al. (32) and Yang et al. (33)

#### 3.3.1 Specialized nursing care

As precision-care paradigms gain traction, clinical practice is moving toward ever-greater specialization and refinement. A Chinese scholar embedded an evidence-based clinical pathway for enterostomy patients into the hospital’s existing NIS and, by exploiting core functions, doubled adherence to critical nursing interventions (13). Similar gains have been observed when nurses confronted the mismatch between generic NIS templates and specialty-specific workflows. In the exploration domain, cardiovascular and hepatobiliary units have developed bespoke NIS modules and “smart wards” that integrate continuous monitoring and AI-driven risk alerts (14); parallel initiatives have introduced specialty nursing consultation models that transcend traditional care boundaries (15). These examples illustrate how exploitation of latent functions and exploration of novel solutions jointly advance specialized practice. Nevertheless, enhancing NIS adaptability to diverse specialty contexts remains an ongoing imperative for nurse-led innovation.

#### 3.3.2 Nursing management

##### 3.3.2.1 Human-resource management

Effective staffing mitigates workforce imbalances and elevates managerial efficiency (16). Building on basic e-rostering functions, charge nurses have combined modules for job-description queries, shift reminders, individual preferences and emergency deployment to achieve personalized, data-driven schedules (17). Furthermore, Knox et al. (18) from the United States conducted in-depth mining and analysis of the data characteristics of the NIS usage (such as the number of patients, the quantity of medication administered, the duration, the start time, etc.) during the peak period of daily medication administration by nurses, and innovatively exploited and summarized a simple and effective system for nurse staffing and workload assessment. It provides new ideas and powerful references for promoting the optimization of human resources. Future managers should continue to leverage the rich, underutilized datasets within NIS to refine staffing decisions.

##### 3.3.2.2 Standardized training and assessment for nurses

Standardized training and assessment management for nurses can not only enhance their professional capabilities but also, to a certain extent, reduce their burden. Zhao et al. (19) from China achieved a breakthrough and innovative attempt in the standardized training of nurses by integrating virtual reality technology, making the teaching of basic nursing operations in the NIS more immersive and gamified. Zhou et al. (20) from China also integrated training resources such as nursing operation norms into the NIS and set up a more flexible, autonomous and diverse assessment mechanism for nurses based on the system. By exploiting the new nursing technology management module of the NIS, nursing managers can manage the standardized training and assessment of nurses based on the feedback data. Therefore, how to solve the problems existing in the standardized training and assessment of nurses in the NIS, such as dull content, single assessment, and insufficient management module architecture, is an important direction for future innovative usage behavior (21).

##### 3.3.2.3 Workflow and quality management

Streamlined workflows and robust quality control are cornerstones of effective nursing administration. In the exploration domain, based on the existing NIS, nurses have innovatively developed a series of scientific, efficient and practical process management systems and nursing quality control systems (22, 23). Exploiting existing functionality, Agnihotri et al. (24) from Canada embedded the Canadian Triage and Scale into an emergency NIS, significantly improving triage accuracy and efficiency. Zhu et al. (25) from China used lightweight Access programming to create bespoke modules that automated planning, implementation and evaluation of departmental activities, thereby enhancing managerial throughput. Wang et al. (26) from China integrated an unplanned-extubation-risk calculator with visual analytics, elevating both risk awareness and quality outcomes. Despite these advances, further functional exploitation is required to achieve comprehensive workflow optimization.

#### 3.3.3 Internet-enabled nursing services

##### 3.3.3.1 Home-based care

The “Zheli Nursing” program has demonstrated the feasibility of a tertiary-hospital-led, tiered-care alliance in China. After mastering the core functions of their hospital NIS, nurses integrated Internet of Things, 5G telecommunication, and human–computer interaction technologies to co-develop home-care platforms such as Healthcare at Home, Internet Plus Care, Phoenix Nurse, and Orange One Nurse (27, 28). These platforms consolidate multidisciplinary resources and deliver online consultation, appointment scheduling, home visits, rehabilitation, and follow-up, expanding nurses’ scope of practice while providing convenient, high-quality care to patients at home.

##### 3.3.3.2 Nurse-led outpatient clinics

Specialty nurse-led outpatient clinics have emerged as an innovative extension of professional nursing. In China, leveraging the existing NIS, Yu et al. (29) established a pediatric epilepsy clinic that delivers sustained internet-based follow-up. Liu et al. (30) created a women- and children-focused tele-clinic, enabling remote assessment and flexible service delivery. Building on a case-management NIS, Lyu and Shen (31) piloted three chronic-disease case-management clinics, optimizing hospital-to-community continuity and yielding measurable improvements. This diversity of models reflects a progression from utilizing NIS for routine digital follow-up toward integrated case-management platforms that prioritize hospital-to-community continuity. Such a shift underscores that the depth of NIS innovation is intrinsically linked to the complexity of the service model, effectively evolving the nurse’s role from providing functional support to coordinating longitudinal, multidisciplinary care.

##### 3.3.3.3 Virtual wards

Unlike traditional inpatient units, virtual wards aggregate geographically dispersed patients into a single digital care environment (32). In a study of 21,281 postoperative patients, Yang et al. (33) showed that a pain-focused virtual ward achieved superior analgesia and reduced adverse-event rates compared with standard care. Within the virtual ward, nurses—supported by the NIS—provide 24-h remote monitoring, assessment and health education as core members of multidisciplinary teams. This approach alleviates bed shortages and extends high-quality postoperative care. Encouraging nurses to innovate on existing NIS platforms is therefore essential for scaling virtual wards globally.

### 3.4 Assessment tools

#### 3.4.1 Information system use behavior scale

Originally developed by Saga and Zmud (34) for the field of enterprise management, the Information System Use Behavior Scale was translated and culturally adapted for Chinese nurses in 2017 (9). The instrument comprises two subscales—exploitation and exploration—in a total of seven items rated on a 5-point Likert scale (1 = strongly disagree to 5 = strongly agree). Total scores range from 7 to 35, with higher scores indicating more pronounced innovative use behaviors. Cronbach’s *α* coefficients for the overall scale and the two subscales are 0.918, 0.904 and 0.922, respectively.

#### 3.4.2 Nurses’ innovative behavior scales

##### 3.4.2.1 The nurses’ innovative behavior scale

The scale was developed by Bao et al. (11), contains 10 items distributed across three dimensions: idea generation, support acquisition and idea implementation. Each item is rated on a 5-point Likert scale (1 = never to 5 = very frequently), yielding a total score of 10–50; higher scores denote greater innovative behavior. Cronbach’s *α* for the overall scale is 0.879 (subscales: 0.746–0.870).

##### 3.4.2.2 The innovative behavior inventory

IBI inventory was originally created by Lukes et al. (35) for business contexts. The 20-item scale covers five dimensions: idea generation and scanning, planning and implementation, resource acquisition, barrier removal and clinical application. Items are rated from 1 (strongly disagree) to 5 (strongly agree), producing a total score of 20–100. Cronbach’s *α* for the overall scale is 0.923 (subscales: 0.779–0.863).

#### 3.4.3 Comparative summary of assessment tools

A comparison across these studies reveals that no single tool perfectly addresses the intersection of clinical nursing and digital innovation. While the IBI provides the most comprehensive process-oriented evaluation, its roots in enterprise management may overlook the high-risk nuances of clinical nursing workflows. Conversely, the Nurses’ Innovative Behavior Scale captures professional traits but lacks the informatics specificity required to measure technical NIS adaptations. Furthermore, while the NIS Use Behavior Scale directly addresses the “exploitation-exploration” framework, its current application is heavily geographically skewed toward China (2), necessitating broader international validation to ensure global evidence comparability.

### 3.5 Determinants

#### 3.5.1 Individual factors

**Figure 1:**
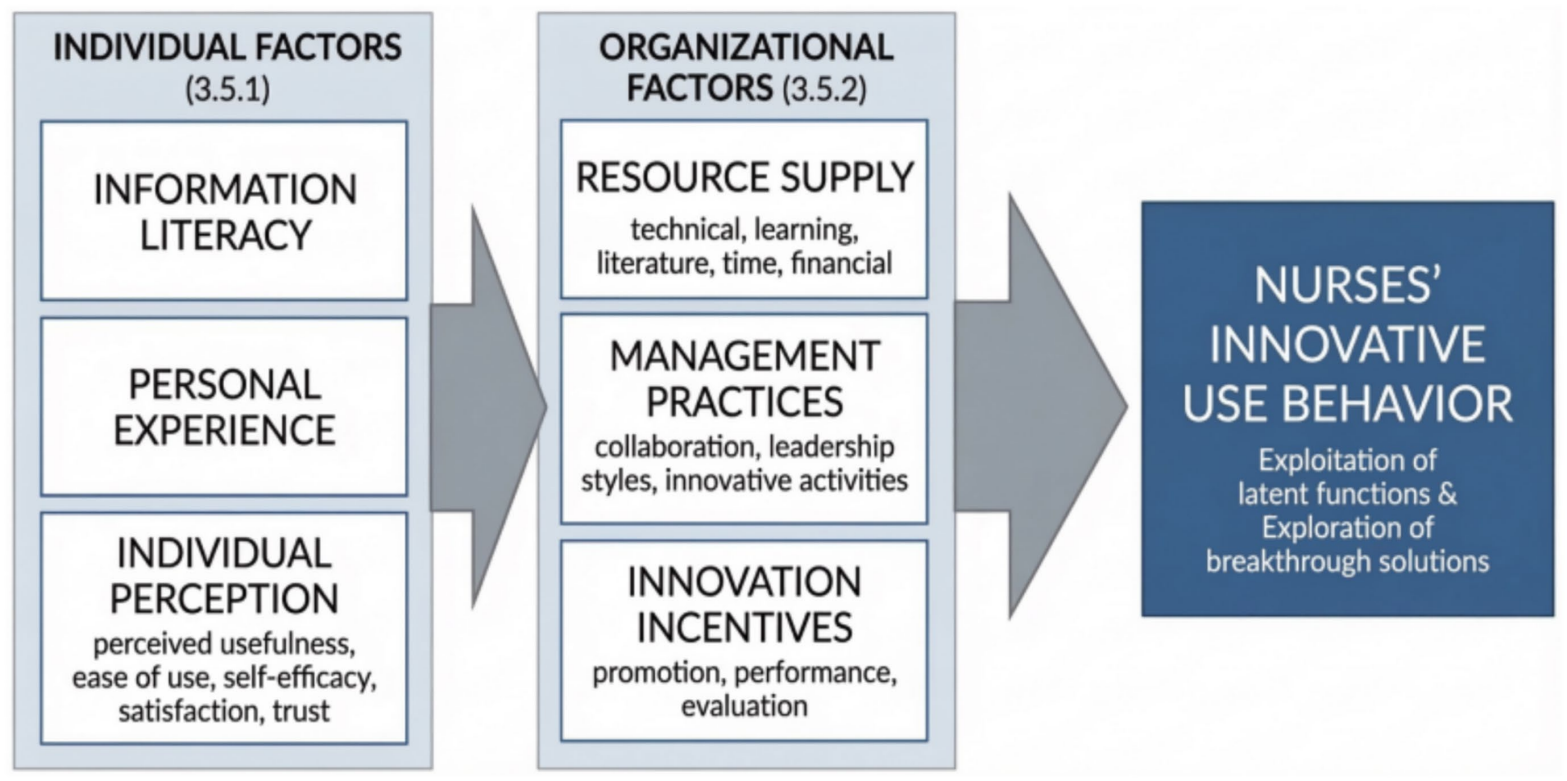
Determinants of nurses’ innovative use behavior of NIS.

Personal factors include nurses’ information literacy, personal experience and individual perception (Figure 1). Information literacy refers to the ability of nurses to identify, acquire, evaluate and utilize information in their nursing work (36). An individual’s information literacy can directly influence their innovative behavior and show a positive trend. The higher the information literacy of nurses is, the more acutely they can detect potential problems in the NIS and propose solutions through innovative usage behaviors. As the driving force for nurses to generate innovative behavior, the improvement of information literacy can help nurses cultivate an information awareness of being good at observing and identifying problems in their nursing work. To better solve problems, nurses often voluntarily make use of new tools, new technologies, etc., thereby achieving innovative application of the NIS. Secondly, the personal experience of nurses, especially those who have received training in the operation of NISs (2), has a significant impact on their innovative usage behavior. Standardized training can help nurses master the core functions of the system and provide them with knowledge reserves for innovative use. In addition, the experience accumulated by nurses in their daily work can also prompt them to apply the system functions more flexibly and explore new usage methods. Combining their own information literacy and innovative ideas, these experiences can prompt nurses to relieve stress and anxiety during the process of using the NIS, thereby providing possibilities for the innovative use of the NIS (6, 37). Finally, individual perception plays a significant role in the use of information systems, covering multiple aspects such as perceived usefulness, perceived ease of use, perceived self-efficacy, satisfaction, and trust. These perceptual factors not only affect users’ initial acceptance of information systems but also have a significant impact on their deep-seated usage behaviors (5). According to the Technology Adoption Model (TAM), perceived usefulness and perceived ease of use are among the key factors for nurses to accept the care information system. When nurses perceive that the information system can effectively enhance work efficiency and the quality of care, and is simple and easy to operate, they are more willing to accept and frequently use the system, thereby promoting its innovative application. The User Value Acceptance Model (VAM) places greater emphasis on nurses’ perception of self-efficacy. When nurses perceive that they have sufficient ability to use the NIS, they are more inclined to actively explore and innovatively use the system. Similarly, the Integrated Technology Acceptance Model (UTAUT) points out that satisfaction and trust are also important factors influencing users’ continuous use of information systems. When nurses are satisfied with the use of the NIS and consider it reliable and safe, they are more likely to make in-depth use of the information system, thus forming a virtuous cycle.

#### 3.5.2 Organizational factors

Organizational factors include resource supply, management practices and innovation incentives (Figure 1). Medical institutions should not only provide nurses with NIS, but also offer supporting supplies such as technical resources, learning resources, literature resources, time resources and financial resources (2), to ensure the innovative usage behaviors of nurses. Internationally, the research and development levels of NIS vary significantly, and there are problems of poor quality of some technical resources (38), which hinders the in-depth mining of nursing data and increases the usage burden on nurses. Similarly, in cases where the supply of other resources is difficult or insufficient, the system is hard to be fully utilized by non-information professional nurses, thereby hindering the advancement of innovative usage behaviors. Management practice refers to the management measures adopted by medical institutions at the organizational level (2). Nursing management practices characterized by cross-disciplinary team collaboration, transformational leadership styles, nurse-led innovative activities, and transparent management mechanisms (39) can enhance the satisfaction of nursing work and team collaboration awareness and create a favorable organizational environment for nurses’ innovative usage behaviors. Based on adequate resource supply and good management practices, innovation incentives are an important driving force for nurses to develop innovative usage behaviors of NIS. The innovative demonstrations and assistance provided by leaders and colleagues, as well as scientific and feasible incentive strategies at the management level (40), can foster a good sense of team innovation among the nurse group, stimulate the innovative enthusiasm and motivation of individual nurses, and enable them to take action, which can effectively promote the innovative use of the NIS by nurses.

In summary, while individual literacy serves as the essential foundation, current evidence suggests that in the absence of organizational empowerment, high individual competence does not automatically translate into innovative behaviors.

### 3.6 Strategies to enhance innovative use

#### 3.6.1 Improve the assessment tool system and build a multi-dimensional measurement framework

In view of the limitations of existing evaluation tools in concept definition, cross-cultural debugging and item expression, a multi-dimensional and dynamic measurement framework needs to be established. Firstly, it is suggested to integrate the advantages of the existing scales (9–11), and combine the professionalism of the nurse’s working scenarios and the particularity of the NIS to develop a hybrid assessment tool. Specifically, this can be achieved through interdisciplinary collaboration, involving clinical nurses, nursing managers, and information technology experts etc. Based on nursing practice cases, the operational concept of “innovative usage behavior” can be redefined, the item expression optimized, and the comprehensibility and practical orientation of the scale enhanced. Secondly, promote the cross-cultural adaptation and validation of assessment tools. Through international multi-center studies, compare the differences in usage behaviors among nurse groups in different countries, clarify the impact of cultural factors on the validity of items, and enhance the universality of the tools. In addition, objective indicators such as behavioral log analysis and system background data tracking can be introduced as supplements to form a composite assessment model of “subjective scales + objective data”, to more comprehensively reflect the dynamic process of nurses’ innovative behavior.

#### 3.6.2 Strengthen the cultivation of nurses’ information literacy and innovation ability, and consolidate the individual foundation

Given the crucial roles of information literacy, experience accumulation and self-perception among personal factors, it is necessary to establish a hierarchical and progressive ability cultivation system. Firstly, nursing managers can collaborate with the hospital’s information department to select dual-qualified compound talent mentors, establish a step-by-step training mechanism for nursing information capabilities, and offer advanced course contents such as module architecture, data extraction, artificial intelligence, and software development. Based on the explicit and implicit indicators such as the clinical department, length of service, information literacy status, and innovation potential value of nurses, differentiated and customized learning content and paths are intelligently pushed and provided (6, 41), and actual cases and simulated scenarios are introduced (42) to deepen the cultivation of nurses’ innovation ability. Secondly, beyond the curriculum, managers can also establish an “innovation case library” and an experience sharing platform to encourage nurses to transform their innovative practices in daily work into reusable standardized solutions. These can be promoted through in-hospital workshops, academic conferences, and other means, thus forming a virtuous cycle of experience accumulation and knowledge dissemination. Finally, based on models such as the technology acceptance model and the user value acceptance model, humanized interaction functions are integrated into the design of the NIS to reduce the complexity of use. At the same time, activities such as “Innovation Achievement Exhibition” and “Benchmark Nurse Selection” are carried out to enhance nurses’ confidence in innovation and stimulate their internal drive for active exploration (41).

#### 3.6.3 Optimize the organizational support environment and establish an incentive mechanism for collaborative innovation

At the organizational level, support should be provided from three aspects: resource guarantee, management innovation and institutional incentives. Firstly, as the backing for nurses’ innovative use of the NIS, medical institutions should start to establish a complete resource guarantee system for the NIS, set up special funds for the upgrade of the NIS and the development of supporting resources, purchase targeted learning and literature libraries to help nurses master the latest information technology and nursing practices, and allocate flexible working hours for innovative projects Optimize the nursing shift scheduling system or set up dedicated positions to create more time resources for nurses (43). Secondly, hospitals can implement an innovative management model of “nurse-led and cross-disciplinary collaboration”, form multi-disciplinary innovation teams composed of nurses, information engineers, etc., and regularly conduct demand research and system optimization iterations. Implement transformational leadership strategies and encourage nursing managers to provide a psychologically safe environment for nurses’ innovative behavior through empowerment and fault-tolerance mechanisms (42). Finally, nursing managers should also establish a long-term policy mechanism that emphasizes both material and spiritual incentives. For instance, they can incorporate innovative behavior into performance evaluations, set up a “Nursing Informatization Innovation Fund” to support the implementation of feasible plans, cultivate an open, transparent and innovation-supporting organizational culture, and at the same time enhance nurses’ sense of gain through in-hospital recognition and revenue sharing from the transformation of academic achievements. Ultimately, a closed-loop drive of “individual innovation—organizational support—outcome feedback” is formed (43).

## 4 Discussion

While this study provides a preliminary conceptual framework for nurses’ innovative use behavior of NIS, the lack of global consensus on definitions remains a primary barrier to standardizing evaluation criteria. From a practical perspective, nurses’ innovative behavior exhibits a step-wise progression from “exploitation” to “exploration.” The exploitation dimension focuses on mining existing system potential to resolve immediate clinical bottlenecks, such as utilizing barcode data for workload assessment or embedding evidence-based clinical pathways to enhance nursing adherence (2, 3, 9). Conversely, the exploration dimension exerts a more profound impact, where nurses integrate emerging technologies like VR for training or construct entirely new service models such as virtual wards, facilitating a role transformation from passive tool users to system co-designers (19, 33). This evolution from simple to complex not only provides concrete guidelines for clinical practice but also helps mitigate long-standing cognitive biases.

However, a critical evaluation of the current evidence strength is essential. Among the 37 included studies, system design and clinical application reports (*n* = 15) predominate. While these studies demonstrate the diverse possibilities for nurse-driven innovation, much of the current literature remains in the “proof-of-concept” stage and lacks rigorous long-term longitudinal empirical research. Consequently, while we observe active system adaptation and innovation by nurses, it remains uncertain whether these behaviors effectively reduce patient mortality or decrease postoperative complications in the long term.

A notable limitation of this review is the geographic concentration of the included evidence, with a predominant focus on China (*n* = 24). This trend likely reflects the rapid digital transformation of the Chinese healthcare system, catalyzed by national policies such as the “Internet + Healthcare” initiatives. While these studies provide a rich blueprint for NIS innovation, their generalizability to broader international contexts may be constrained due to differences in nursing-to-patient ratios, legal frameworks, and institutional cultures.

Furthermore, limitations in current research designs hinder the advancement of scientific depth. Most existing studies utilize single-center, small-sample designs, which significantly limits the generalizability of the findings and makes it difficult to apply them directly to healthcare environments with different resource allocations or cultural backgrounds. To achieve precise intervention, future research should develop multi-dimensional assessment models that combine subjective scales with objective background log data to more comprehensively capture the dynamic process of innovation.

Finally, the integrated analysis of individual literacy and organizational resources indicates that improving nurses’ innovative NIS utilization cannot rely on a single dimension. Rather, it requires a synergistic strategy combining “bottom-up” individual empowerment with “top-down” organizational incentives. This systematic improvement pathway provides clear directional recommendations for the deep transformation of nursing informatization in the future.

## 5 Implications for nursing and health policy

To foster a sustainable environment for NIS innovation, health policy focus might consider transitioning from basic digital literacy toward structural empowerment. The shift from passive compliance to proactive exploration suggests that policymakers should encourage clinical nurses to serve as co-designers in system development to ensure tools align with clinical realities. Institutionally, providing specific resources—such as innovation funds or protected research time—may be necessary to allow nurses to adapt systems to specialized needs, such as virtual ward management. Furthermore, integrating innovative use behaviors into professional advancement and performance evaluations could offer a more consistent incentive for professional growth. Finally, addressing interoperability and resource equity is suggested to ensure that digital advancements benefit nurses across diverse healthcare settings, including those in low-resource environments.
