# Supplementary material for: Nurses’ innovative use of information systems: a narrative review
Source: Front Public Health. 2026 May 25;14:1808207. doi: 10.3389/fpubh.2026.1808207 (PMC13243254; doi:10.3389/fpubh.2026.1808207)
Supplement: Supplementary file 1 [file Data_Sheet_1.PDF]

Appendix1

1. Table A1: Search Strategy and keywords ------------------------------- Page 1
2. Table A2: Inclusion and Exclusion Criteria ---------------------------- Page 2-3
3. Figure A1: Flowchart for study search and screening -------------------- Page 4
4. Table A3: Full Characteristics of Included Studies --------------------- Page 5-9
5. SANRA checklist -- Page 10

■ **Table A1: Search Strategy and keywords**

Item	Category	Search Keywords
#1	Nurse	nurs*[Title/Abstract]
Ω<br>#2	Nursing Information Systems	information system*[Title/Abstract] OR<br>system*[Title/Abstract] OR<br>platform*[Title/Abstract] OR<br>software[Title/Abstract] OR<br>application*[Title/Abstract]
#3	Innovative Usage Behavior	innovat*[Title/Abstract] OR<br>exploitat*[Title/Abstract] OR<br>explorat*[Title/Abstract] OR<br>customiz*[Title/Abstract] OR<br>optimiz*[Title/Abstract] OR<br>adapt*[Title/Abstract] OR<br>use behavio*[Title/Abstract] OR<br>usage behavio*[Title/Abstract]
#1 AND #2 AND #3		

■ **Table A2: Inclusion and Exclusion Criteria**

Item	Inclusion Criteria	Exclusion Criteria
Population	☑Registered nurses (RNs)<br>☑Nurse managers<br>☑Nurse practitioners<br>☑nursing students	☐Non-nursing professionals<br>☐Purely computer technology developers
Interest	☑Nursing Information Systems (NIS): Defined as a computer system that collects, stores, processes, retrieves, displays, and communicates timely information needed to support nursing practice, administration, education, and research.	☐General hospital administrative software that does not involve nursing-specific data or workflows.
Context	☑Studies describing nurses' use of NIS beyond routine operational functions:<br>1. **Exploitation**: The context that focus on nurses proactively mastering and leveraging existing NIS functions, as well as discovering and combining latent system capabilities to optimize clinical workflows and specialized care pathways.<br>2. **Exploration**: The review incorporates research highlighting nurses' integration	☐Studies focusing solely on routine system usage without exploring optimization, behavioral factors, or development processes.<br>☐Technical papers focused on back-end coding without clinical nursing involvement.

	of emerging technologies to develop new software modules or implement breakthrough nursing service models that transcend the system's original functional framework.	
Study Type	☑Original empirical research: Quantitative, Qualitative, or Mixed-Methods<br>☑Review<br>☑Academic dissertations/ theses	☐Editorials<br>☐Commentaries<br>☐Book reviews<br>☐Opinion pieces<br>☐Conference abstracts
Language	☑Chinese<br>☑English	☐Other Languages
Data Integrity	☑The full text can be obtained and the data results are clear and complete.	☐Literature that cannot be obtained in full text, has been published repeatedly, or is severely lacking in data.

■ Figure A1: Flowchart for study search and screening.

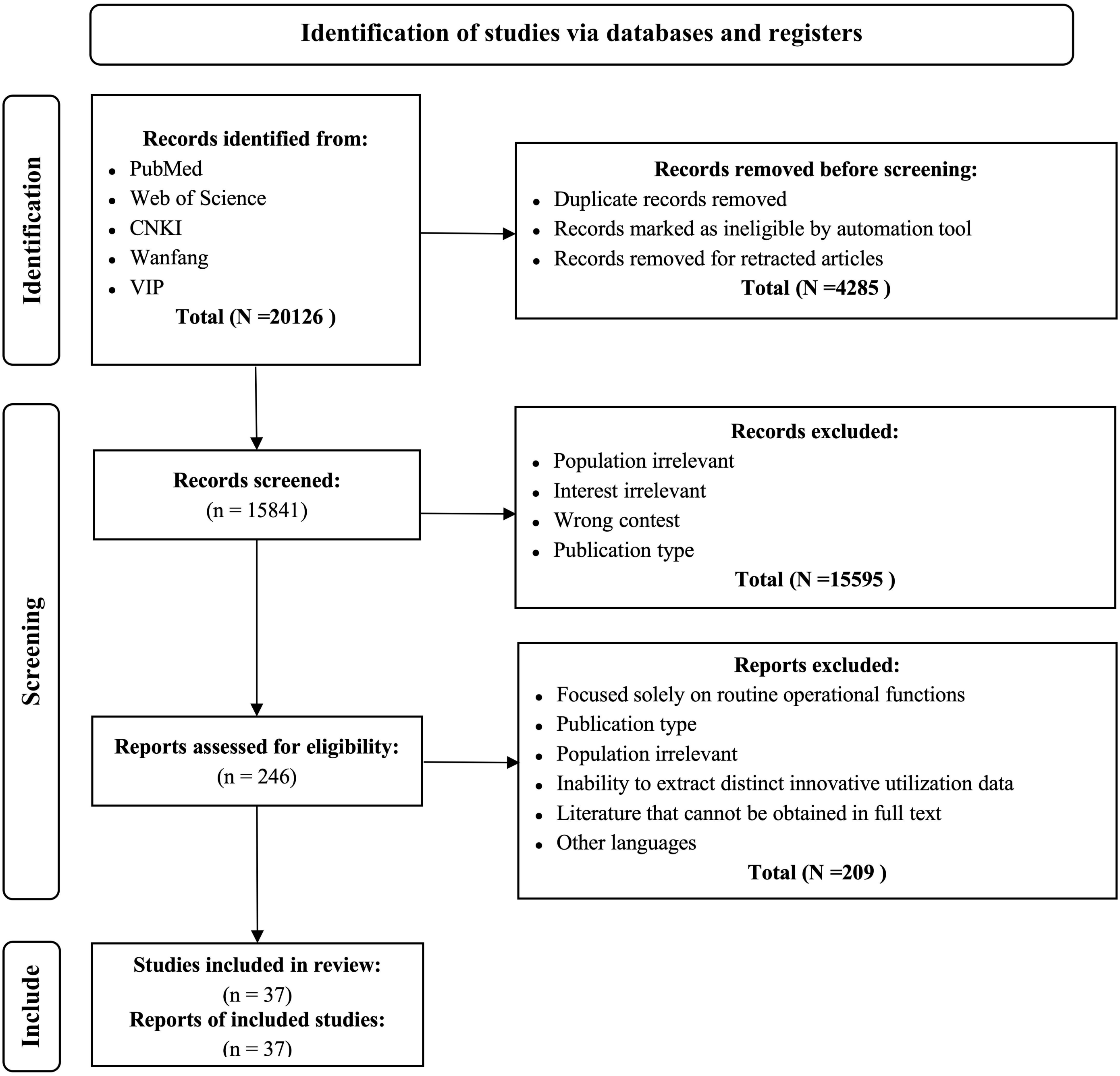

Note: The initial results were 20126 items. After duplicate removal and screening by title, abstract, and full text, a total of 37 papers were included for final analysis

■ **Table A3: Full Characteristics of Included Studies (N=37)**

ID	Author (Year)	Country/ Setting	Study Design	Synthesis Category	Key Findings/ Innovative Behaviors
1	Wu H (2021)	China	Master Thesis	Conceptualization; Assessment tools; Determinations	Identified factors influencing innovative NIS use and validated relevant assessment items.
2	Meng L (2020)	China	Master Thesis	Conceptualization	Research on Influencing Factors of Innovative Use Behavior of Enterprise Information Systems.
3	Hashish (2025)	Saudi Arabia/ Egypt	Qualitative Study	Conceptualization	Investigated E-leadership competencies and digital transformation perspectives.
4	Lin IC (2016)	Taiwan, China	Empirical Study	Conceptualization; Determinations	Evaluated usage behavior stability and perceived usefulness in clinical nursing.
5	Kaihlanen (2023)	Finland	Latent Profile Analysis	Conceptualization; Determinations	Linked informatics competence with perceptions of system usefulness.
6	Walzer S (2025)	Germany	Umbrella Review	Conceptualization	Systematized factors influencing implementation and adoption of digital nursing.
7	An B	South	Bibliometric	Conceptualization	Analyzed global trends in

	(2026)	Korea	Analysis		digital technology application within nursing.
8	Wen X (2017)	China	Scale Validation	Conceptualization; Assessment tools	Validated the Chinese version of the NIS Use Behavior Scale.
9	Huang L (2021)	China	Scale Validation	Conceptualization	Tested the reliability and validity of the Innovative Behavior Scale (IBI) for nurses.
10	Bao L (2012)	China	Scale Development	Conceptualization; Assessment tools	Developed the Nurse Innovative Behavior Scale (10 items, 3 dimensions).
11	Pei C (2024)	China	System Application	Current practice	Embedded evidence-based clinical pathways for enterostomy into NIS.
12	Liu X (2021)	China	System Design	Current practice	Built AI-driven risk alerts and consultation platforms in hepatobiliary units.
13	Guo Y (2023)	China	System Design	Current practice	Constructed information platforms for specialized nursing consultation.
14	Nantsupawat (2022)	Thailand/ USA/ China	Cross-sectional	Current practice	Analyzed NIS-driven staffing data and its impact on quality of care.
15	Shen Z (2020)	China	Review	Current practice	Synthesized progress in nursing management informatization in China.
16	Knox MK	USA	Empirical	Current practice	Mined barcode medication

	(2023)		Study		data to assess staffing and workload.
17	Zhao M (2024)	China	System Design	Current practice	Integrated VR technology into NIS for gamified nurse training.
18	Zhou P (2021)	China	System Design	Current practice	Developed multi-terminal skill management systems for ENT nursing.
19	C B (2023)	Crema Italy/ Italy/ Spain	System Analysis	Current practice	Evaluated transformation from EHR to clinical management systems.
20	Dai Y (2024)	China	System Design	Current practice	Developed intelligent transfusion systems based on photoelectric technology.
21	Wan W (2021)	China	System Design	Current practice	Created multi-terminal nursing quality management systems.
22	Agnihotri T (2021)	Canada	Workflow Analysis	Current practice	Embedded electronic decision-support into the nursing triage process.
23	Zhu X (2020)	China	System Design	Current practice	Used Access databases to automate departmental office systems.
24	Wang P (2023)	China	System Design	Current practice	Integrated unplanned extubation risk calculators with clinical decision

					support.
25	Cui L (2024)	China	Empirical Study	Current practice	Built Internet-based platforms for online-offline discharged patient care.
26	Dukhanin V (2023)	USA	Practice Report	Current practice	Co-designed shared access initiatives for older adult patient portals.
27	Yu X (2022)	China	Practice Report	Current practice	Leveraged NIS for pediatric epilepsy nurse-led clinics.
28	Liu M (2021)	China	Practice Report	Current practice	Constructed Women and Children's Internet nursing clinics.
29	Lyu Y (2020)	China	Case Management	Current practice	Promoted case management clinics for chronic disease using NIS.
30	Westby M (2024)	UK	Realist Review	Current practice	Defined "what works" in virtual wards for frail populations.
31	Yang G (2023)	China	Empirical Study	Current practice	Implemented virtual pain units for improved postoperative analgesia.
32	Saga & Zmud (1993)	USA	Theoretical Model	Assessment tools	Developed the original conceptual nature of IT infusion.
33	Lukes (2017)	UK	Review	Assessment tools	Created the Innovative Behavior Inventory (IBI) for cross-cultural use.

34	Jiang R (2023)	China	Empirical Study	Determinations	Studied the impact of information literacy on innovative behavior.
35	Tian Y (2019)	China	Empirical Study	Determinations	Analyzed influencing factors of NIS usage in tertiary hospitals.
36	Hashemi N (2020)	Iran	Viewpoint Study	Determinations	Identified barriers and facilitators to HIS use from nurses' perspectives.
37	Labrague (2023)	USA	Systematic Review	Determinations	Linked leadership styles to nurses' innovative behaviors.

■ SANRA checklist

Scale for the Assessment of Narrative Review Articles – SANRA

Please rate the quality of the narrative review article in question, using categories 0–2 on the following scale. For each aspect of quality, please choose the option which best fits your evaluation, using categories 0 and 2 freely to imply general low and high quality. These are not intended to imply the worst or best imaginable quality.

1) Justification of the article's importance for the readership

The importance is not justified. ______ 0
The importance is alluded to, but not explicitly justified. ______ 1
The importance is explicitly justified. ______ 2

2

2) Statement of concrete aims or formulation of questions

No aims or questions are formulated. ______ 0
Aims are formulated generally but not concretely or in terms of clear questions. ______ 1
One or more concrete aims or questions are formulated. ______ 2

2

3) Description of the literature search

The search strategy is not presented. ______ 0
The literature search is described briefly. ______ 1
The literature search is described in detail, including search terms and inclusion criteria. ______ 2

1

4) Referencing

Key statements are not supported by references. ______ 0
The referencing of key statements is inconsistent. ______ 1
Key statements are supported by references. ______ 2

1

5) Scientific reasoning

(e.g., incorporation of appropriate evidence, such as RCTs in clinical medicine)
The article's point is not based on appropriate arguments. ______ 0
Appropriate evidence is introduced selectively. ______ 1
Appropriate evidence is generally present. ______ 2

2

6) Appropriate presentation of data

(e.g., absolute vs relative risk; effect sizes without confidence intervals)
Data are presented inadequately. ______ 0
Data are often not presented in the most appropriate way. ______ 1
Relevant outcome data are generally presented appropriately. ______ 2

2

Sumscore **10**
